# Diphtheria Anti-Toxin in Epidemic Measles

**Published:** 1898-01-15

**Authors:** 


### DIPHTHERIA ANTI-TOXIN IN EPIDEMIC MEASLES.

Dr. Northrup, of New York, has been using diphtheria anti-toxin to prevent the occurrence of diphtheria as a complication of measles. He gives an account in the *Medical News* of an outbreak of measles in the New York Foundling Hospital, which attacked 258 cases, causing death in 36. Previous experience had shown that diphtheria was very apt to complicate measles, and it was determined on this occasion to immunise all those who were not believed to be, by reason of their age or location in the hospital, reasonably safe from diphtheria. Of those believed to be in danger of contracting this disease (those aged from 2½ to 4½ years, or located in or near nurseries in which diphtheria had previously been observed) 129 were immunised. Of these 77 caught measles and passed through its stages without contracting diphtheria. In one nursery at the top of the administrative block, and widely separated from the rest, no cases were immunised, and no cases developed diphtheria; but in a second nursery, where diphtheria had been occasionally observed, nine children were not immunised, and the third day after the appearance of the rash four out of the nine had a bloody nasal discharge, which on culture showed diphtheria bacilli. It is worth noting that in two cases which were immunised pharyngeal diphtheria developed after 31 and 33 days respectively, which shows that the immunity obtained was not of long duration. We think that Dr. Northrup's experience is well worth bearing in mind, and that immunisation with diphtheria anti-toxin may be of much service in some cases, both in private and in public practice, where both measles and diphtheria are known to be prevailing together. At the same time it is difficult not to feel that the tendency of diphtheria to recur over and over again in the same dormitories, which, from the account given seems to have been observed in the New York Foundling Hospital, points to something wrong either in the sanitation or the discipline of the rooms in question.

